# Supplementary material for: Non-Hermitian engineering of single mode two dimensional laser arrays
Source: Sci Rep. 2016 Oct 4;6:33253. doi: 10.1038/srep33253 (PMC5048422; doi:10.1038/srep33253)
Supplement: Supplementary Information [file srep33253-s1.pdf]

# Supplementary material

# Non-Hermitian engineering of single mode two dimensional laser arrays

*Mohammad H. Teimourpour[1,2], Li Ge[3,4], Demetrios N. Christodoulides[5] and Ramy El-Ganainy[1,2,*]*

[1]Department of Physics, Michigan Technological University, Houghton, Michigan, 49931, USA

[2]Henes Center for Quantum Phenomena, Michigan Technological University, Houghton, Michigan, 49931, USA

[3]Department of Engineering Science and Physics, College of Staten Island, CUNY, Staten Island, NY 10314, USA

[4]The Graduate Center, CUNY, New York, NY 10016, USA

[5]College of Optics & Photonics–CREOL, University of Central Florida, Orlando, Florida 32816, USA

[*]ganainy@mtu.edu

## A. Householder Method

Householder method for tridiagonalization of matrices was introduced by A. S. Householder in 1985 [1]. It reduces an $n \times n$ symmetric matrix to a similar tridiagonal one by performing $n-2$ orthogonal transformations. This method finds a wide range of applications in linear algebra. The details of how the method works can be found in refs. [1,2]. Here we present the algorithm in MatLab language for given general $n \times n$ symmetric matrix A:

```
[n,n]=size(A);
for k=1:n-2
    X=A(:,k);
    ss=0;
    for j=k+1:n
    ss=ss+X(j)^2;
    end
```

```
    S=sign(X(k+1))*sqrt(ss);
    R=sqrt(2*(S+X(k+1))*S);
    W=X;
    W(1:k)=0;
    W(k+1)=W(k+1)+S;
    W=(1/R)*W;
    V=A*W;
    c=W'*V;
    Q=V-c*W;
    A=A-2*W*Q'-2*Q*W';
end
```

**B. Eigenvalue degeneracy in square arrays**

The eigenstate degeneracy of uniform square arrays can be in general investigated by using the point group $D_4$ [3]. Other symmetry groups have to be considered for arrays having different topologies. However here, and since we focus on square arrays only, we discuss this feature by using an alternative straightforward algebraic method. At lasing threshold $g=\gamma$, equation (1) written in the rotating frame, takes the form: $i\frac{da_{m,n}}{dt}=\left(\kappa_{m,n}^{m-1,n}a_{m-1,n}+\kappa_{m,n}^{m+1,n}a_{m+1,n}\right)+\left(\kappa_{m,n}^{m,n-1}a_{m,n-1}+\kappa_{m,n}^{m,n+1}a_{m,n+1}\right)$. By using separation of variable $a_{m,n}(t)=\varphi_m(t)\eta_n(t)$ and substituting back, it is straightforward to show that:

$$\frac{1}{\varphi_m}\left\{i\frac{d\varphi_m}{dt}-\left(\kappa_{m,n}^{m-1,n}\varphi_{m-1}+\kappa_{m,n}^{m+1,n}\varphi_{m+1}\right)\right\}+\frac{1}{\eta_n}\left\{i\frac{d\eta_n}{dt}-\left(\kappa_{m,n}^{m,n-1}\eta_{n-1}+\kappa_{m,n}^{m,n+1}\eta_{n+1}\right)\right\}=0 \quad \text{(B.1)}$$

In the most general case, the two terms in the brackets cannot be satisfied independently. However, for square arrays with identical rows and identical columns, i.e. $\kappa_{m,n}^{m\pm1,n}=\kappa_{m,n'}^{m\pm1,n'}\equiv T_m^{m\pm1}$ $\kappa_{m,n}^{m,n\pm1}=\kappa_{m',n}^{m',n\pm1}\equiv J_n^{n\pm1}$, the two terms become independent and we obtain:

$$i\frac{d\varphi_m}{dt} = T_m^{m-1}\,\varphi_{m-1} + T_m^{m+1}\,\varphi_{m+1} \qquad \text{(B.2.a)}$$

$$i\frac{d\eta_n}{dt} = J_n^{n-1}\,\eta_{n-1} + J_n^{n+1}\,\eta_{n+1} \qquad \text{(B.2.b)}$$

Evidently if $\mu_m$ and $\mu_n$ are the eigenvalues associated with equations (B.2.a) and (B.2.b), respectively (note that the indices of $\mu$ characterize the different eigenvalues and do not indicate array elements), i.e. $\varphi_m \propto \varphi_o \exp(-i\mu_m t)$ and $\eta_n \propto \eta_o \exp(-i\mu_n t)$, it follows that the eigenvectors of full system satisfy $a_{m,n} = \varphi_m \eta_n \propto \varphi_o \eta_o \exp\left[-i\left(\mu_m + \mu_n\right)t\right]$ and the associated eigenvalues are given by $\mu_{m,n} = \mu_m + \mu_n$. Degeneracies thus occur whenever $\mu_m + \mu_n = \mu_{m'} + \mu_{n'}$ for any integer indices $m, n, m'$ and $n'$.

**Square uniform arrays**

From the above analysis, it is clear that $\mu_{m,n} = \mu_{n,m}$. Also in the absence of any accidental degeneracy, the eigenvalues $\mu_{m,m}$ are unique. In addition, if one writes equations (B.2.a) and (B.2.b) in Hamiltonian forms, i.e. $i\frac{d\vec{\varphi}}{dt} = H_\varphi \vec{\varphi}$ and $i\frac{d\vec{\eta}}{dt} = H_\eta \vec{\eta}$, it is easy to show that $\{\sigma_z, H_{\varphi,\eta}\} = 0$ where $[\sigma_z]_{ij} = \delta_{ij}\left(-1\right)^{i+1}$ and the brackets $\{\ \}$ denote anti-commutation. In other words, the Hamiltonians $H_{\varphi,\eta}$ respect chiral particle-hole symmetry: each positive eigenvalue must be accompanied by a negative eigenvalue [4]. Consequently, the eigenspectrum is symmetric about zero. If the integer $N$ is even, the eigenvalues of $H_{\varphi,\eta}$ do not include any zero value and the null eigenvalues of the 2D system are only of the form $\mu_{m,-m}$. As a result, the system exhibits

$N$ unique eigenvalues of the form $\mu_{m,m}$, $N$ zero eigenvalues and doubly degenerate eigenvalues of the form $\mu_{m,n} = \mu_{n,m}$. The total number of distinct eigenvalues is thus given by $N + 1 + \frac{N^2 - 2N}{2} = \frac{N^2 + 2}{2}$. We illustrate this result by the chart shown in figure B.1 for the $4 \times 4$ array where degenerate eigenvalues having the same value are highlighted by the same color. Similar considerations apply to the case of odd value of $N$, except that we must take into account that here the particle-hole symmetry forces one of the eigenvalues of $H_{\varphi,\eta}$ to be zero and thus results in an additional accidental degeneracy. In this case, the total number of distinct eigenvalues turns out to be $\frac{N^2 + 1}{2}$. By applying these formulas for the two square arrays discussed in section two, we indeed find that the $3 \times 3$ and $4 \times 4$ arrays exhibit five and nine distinct eigenvalues, respectively.

We note that in the above analysis, we have assumed that apart from the zero eigenvalue dictated by the chiral symmetry in the case when $N$ is odd, no other accidental degeneracy arises. Figure. B.1 illustrates these degeneracy for the case of $4 \times 4$ array where only nine distinct eigenfrequencies exist.

| | $\mu_2$ | $\mu_1$ | $-\mu_1$ | $-\mu_2$ |
|---|---|---|---|---|
| $\mu_2$ | $2\mu_2$ | $\mu_1+\mu_2$ | $\mu_2-\mu_1$ | 0 |
| $\mu_1$ | $\mu_1+\mu_2$ | $2\mu_1$ | 0 | $-(\mu_2-\mu_1)$ |
| $-\mu_1$ | $\mu_2-\mu_1$ | 0 | $-2\mu_1$ | $-(\mu_1+\mu_2)$ |
| $-\mu_2$ | 0 | $-(\mu_2-\mu_1)$ | $-(\mu_1+\mu_2)$ | $-2\mu_2$ |

Figure. B.1 Eigenvalue structure of a $4 \times 4$ uniform square array. Nine distinct eigenfrequencies exist as highlighted by the different colors.

**Square bosonic arrays**

The above discussion applies equally to the square bosonic arrays introduced in section 3 in the main text. However, here in addition to the geometric induced degeneracies, accidental degeneracies also occur. In the other words, in bosonic arrays, the condition $\mu_m + \mu_n = \mu_{m'} + \mu_{n'}$ can be satisfied for a set of modes (characterized by the indices $m, n, m'$ and $n'$) that do not necessarily transform into one another under geometric operations such as reflection and rotation. In particular, due to the equidistant eigenvalue ladder of one dimensional bosonic arrays, the degeneracy condition $\mu_m + \mu_n = \mu_{m'} + \mu_{n'}$ in two dimensional configurations holds when $m + n = m' + n'$. By taking these accidental symmetries into account, we find that the total number of non-degenerate eigenstates in an $N \times N$ bosonic array is given by $2N - 1$. Figure. B.2 illustrates these degeneracies for the case of $4 \times 4$ bosonic array where only seven distinct eigenfrequencies exist.

| | $3\Delta$ | $\Delta$ | $-\Delta$ | $-3\Delta$ |
|---|---|---|---|---|
| $3\Delta$ | $6\Delta$ | $4\Delta$ | $2\Delta$ | $0$ |
| $\Delta$ | $4\Delta$ | $2\Delta$ | $0$ | $-2\Delta$ |
| $-\Delta$ | $2\Delta$ | $0$ | $-2\Delta$ | $-4\Delta$ |
| $-3\Delta$ | $0$ | $-2\Delta$ | $-4\Delta$ | $-6\Delta$ |

Figure. B.2 Eigenvalues of a $4\times4$ bosonic array where only seven distinct eigenfrequencies exist. Here we have an equidistant eigenvalue spectrum for $J_x$ laser array and $2\Delta$ corresponds to the eigenvalue ladder steps associated with the 1D Hamiltonians $H_{\varphi,\eta}$.

**C. Absence of degeneracy in 1D discrete systems**

As we have discussed in the main text, the eigenspectra of one dimensional systems do not exhibit any degeneracies. For completeness, we sketch here a simple proof of this known fact. A 1D discrete system is described by a tridiagonal matrix. For any given eigenvalue, the elements of the corresponding eigenvector can be expressed in terms of that eigenvalue, the matrix elements and the first component of that eigenvector which can be chosen arbitrarily. Now assume that there exist two different eigenvectors that correspond to the same eigenvalue. Due to the linearity of the problem, we can scale the first component of the second eigenvector to match that of the first. But as a result, the rest of the components of the eigenvectors will be equal after the scaling. This in turn means that the originally different eigenvectors were related by a constant multiplication factor and hence are basically the same. We note that these restrictions that lead to the impossibility of degeneracy in 1D systems are lifted in higher dimensions where degeneracy is allowed.

**References:**


1. Householder, A.S. Unitary Triangularization of a Nonsymmetric Matrix. Journal of the ACM **5**, 339–342 (1958).
2. Press, W.H., Teukolsky, S.A., Vetterling, W.T. and Flannery, B.P. *Numerical Recipe: The Art of Scientific Computing*, Cambridge University Press; 3rd edition (September 10, 2007).

3. Tinkham, M. Group Theory and Quantum Mechanics (Dover Publications, 2003).
4. Ryu, S. & Hatsugai, Y. Topological Origin of Zero-Energy Edge States in Particle-Hole Symmetric Systems. Phys. Rev. Lett. **89**, 077002 (2002).
